# Gender-specific linkages of parents’ childhood physical abuse and neglect with children’s problem behaviour: evidence from Japan

**DOI:** 10.1186/s12889-016-3072-3

**Published:** 2016-05-14

**Authors:** Takashi Oshio, Maki Umeda

**Affiliations:** Institute of Economic Research, Hitotsubashi University, 2-1 Naka, Kunitachi-shi, Tokyo, 186-8603 Japan; Graduate School of Nursing Science, St. Luke’s International University, 10-1 Akashi-cho, Chuo-ku, Tokyo, 104-0044 Japan

**Keywords:** Childhood abuse, Problem behaviour, Psychological distress

## Abstract

**Background:**

Childhood abuse has far-reaching effects, not only for survivors of maltreatment but also for subsequent generations. However, the mechanism of such intergenerational linkages has not been fully explored. This study investigated this linkage with special reference to its gender-specific features.

**Methods:**

A dataset of parents and their children, obtained from a cross-sectional survey in the Tokyo metropolitan area of Japan, was used. The study sample consisted of 1750 children aged between 2 and 18 years (865 daughters and 885 sons) and their parents (1003 mothers and fathers). Regression models were estimated to assess the associations among 1) both parents’ childhood physical abuse and neglect (childhood abuse), 2) parents’ psychological distress, as measured by the Kessler Psychological Distress Scale (K6), and 3) children’s problem behaviour, as measured by the clinical scales of the Child Behavior Checklist.

**Results:**

Daughters’ problem behaviour was more closely associated with mothers’ than fathers’ childhood abuse, whereas sons’ problem behaviour was more closely associated with their fathers’ experience. The impact of mothers’ childhood abuse on daughters’ problem behaviour was mediated at a rate of around 40 % by both parents’ psychological distress. The proportion of the effect mediated by parents’ psychological distress was less than 20 % for the impact of fathers’ childhood abuse on sons’ problem behaviour.

**Conclusion:**

The intergenerational impact of parental childhood abuse on children’s problem behaviour is gender specific, i.e. largely characterized by the same gender linkages. Further studies that explore the mechanisms involved in the intergenerational impact of childhood abuse are needed.

## Background

Childhood abuse is one of the most important issues that must be addressed in public health, because of the magnitude of its negative impact across generations [1]. It is widely recognized that the experience of having been abused in childhood increases the risk of problem behaviour in the offspring of abuse victims [2–4]. Poor mental health among those who experienced abuse in childhood may contribute to the intergenerational linkage between individuals’ childhood abuse and problem behaviour in their offspring. This pathway has been suggested by studies that found significant associations of parents’ poor mental health with their childhood experience of being abused [5–8] and their offspring’s problem behaviour [9–11].

The association between parental childhood abuse and children’s problem behaviour may differ across parent–child dyads. The theory of same-gender dyads suggests that parents’ psychological and behavioural properties would be represented more strongly among children of same gender, and would, thus, have a greater influence on the development of children of the same gender [12, 13]. Unless these potential gender-specific relationships are considered, any observed association between parental childhood abuse and children’s problem behaviour would be misleading, in that it may reflect only averaged gender-specific relationships, which are not very informative.

Previous studies found that maternal psychological distress partially explained the association between mother’s experience of being abused and their children’s externalizing [3] and internalizing [4] behaviour. In contrast, little is known about the contribution of fathers’ poor mental health to the paternal linkage between childhood abuse and children’s problem behaviour. Most studies of parental influence on children’s problem behaviour have focused exclusively on mothers, assuming that mothers’ greater role as primary caregivers means that they have the strongest effect on children’s psychosocial development [2–4, 9–11]. The mediating effect of psychological distress, thus, may be more limited for fathers than mothers, given that fathers’ commitment to childcare is generally lower than mothers’ especially in a Japanese context [14].

Because these issues have not been fully explored in previous studies, we investigated the intergenerational association between parents’ childhood abuse and children’s problem behaviour using a cross-sectional dataset of people in Japan with special reference to its gender-specific features. To this end, we first examined maternal and paternal linkages between childhood physical abuse/neglect (childhood abuse) and children’s problem behaviour. Second, we examined the mediating effect of mothers’/fathers’ psychological distress on the association between their own experience of childhood abuse and their children’s behavioural problems. In these analyses, we investigated four types of parent–child dyads (mother–daughter, mother–son, father–daughter, and father–son) to determine how sensitive the intergenerational impact of parental childhood abuse is in relation to these combinations.

## Methods

### Study sample and procedure

We used data from the Japanese Study of Stratification, Health, Income, and Neighborhood (J-SHINE) [15]. The survey was conducted in four municipalities in the Tokyo metropolitan area of Japan, with a probabilistic sample of community-dwelling men and women aged 25–50 years. The first-wave survey was conducted in 2010 and 4359 people participated (response rate: 51.8 %). The surveys for the respondents’ spouses (1873) and children (2612) were conducted in the same year (response rates: 61.9 and 67.7 %). The spouse survey was responded to by spouses, and the children survey was responded to by mothers (in most cases) or other caregivers. The second-wave surveys for respondents (2961), spouses (1799), and children (2428) were conducted in 2012 and 2013, with the almost the same questionnaire. In the current study, we used the information in the second-wave survey for the families who participated in both first- and second-wave surveys or only in the second-wave survey, and the information in the first-wave survey for those who only participated in that survey.

We concentrated on couples who were both in their first marriage and residing with their biological child(ren), and limited the analysis to those couples with children aged 2–18 years. In addition, we excluded respondents and their family members who did not report relevant information needed for statistical analysis. As a result, we used the data of 1003 couples and their 1750 children (865 daughters and 885 sons), which consisted of 71.6 % of couples with child(ren) in the original sample.

### Measures

#### Childhood physical abuse and neglect (childhood abuse)

We utilized the reported answers (yes or no) to questions about experiences of parental physical abuse and neglect before graduating from junior high school at age 15: ‘Were you often pushed, did you have an object thrown you, or were you hit by either of your parents?’ (physical abuse) and ‘Did your parents often fail to provide necessary care, such as giving you three meals a day, medical treatment, and other daily necessities?’ (neglect). Respondents were considered to have experienced childhood abuse if they reported at least one instance of physical abuse and neglect.

### Children’s problem behaviour

We focused on two aspects of children’s problem behaviour: internalizing and externalizing, based on the Japanese translations of the parent-reported Child Behavior Checklist (CBCL) for children aged 2–3 years (CBCL/2–3) and aged 4–18 years (CBCL/4–18), [16] which have been validated with Japanese data [17]. CBCL items were first scored on eight narrow-band syndrome scales using a 3-point scale (0 = not true; 1 = somewhat/sometimes true; 2 = very/often true), and then broad-band internalizing and externalizing problem scores were calculated. Next, CBCL scores were divided into three ranges (normal, borderline, and clinical) for internalizing and externalizing behaviour, respectively, based on the established procedure. Finally, we constructed binary variables, allocating 1 to the clinical range and 0 to others for each behaviour.

#### Parents’ psychological distress

We measured psychological distress using the Kessler Psychological Distress Scale (K6) [18]. Respondents were asked to answer a six-item psychological distress questionnaire—‘During the past 30 days, about how often did you feel a) nervous, b) hopeless, c) restless or fidgety, d) so depressed that nothing could cheer you up, e) that everything was an effort, and f) worthless?’—on a five-point scale (0 = *none of the time* to 4 = *all of the time*). Then, the sum of the reported scores (range: 0–24) was calculated and defined as the K6 scale score. Higher K6 scale scores reflect higher levels of psychological distress. K6 scale scores ≥5 indicate the presence of a mood/anxiety disorder in a Japanese sample, as validated by Sakurai et al. [19]. We used a binary variable for psychological distress, which was constructed by allocating 1 to K6 scale scores ≥5 and 0 to other scores.

#### Covariates

As regards educational attainment, we constructed a binary variable for lower educational attainment (graduated from high school or below) for each parent. Regarding household income, respondents selected their household income from 15 income bands. We calculated a median for each band and equivalized the income by dividing by the root of the number of household members. This adjustment calculation is based on recent publications of the Organisation for Economic Co-operation and Development [20]. Then, we constructed a binary variable for household income poverty, by allocating 1 to households with an income in the lowest quartile and 0 to other income bands.

We also introduced two indicator variables related to CBCL in the regression models. The first was the indicator for children aged 4–18 years, considering that the content of the CBCL questionnaire differs between the two age groups. The second was the indicator for a mother responding to the children survey, considering the potential bias owing to differences in the relationship with the child. As covariates, we considered parents’ educational attainment and household income.

### Statistical analyses

We first examined the impact of childhood abuse on parents’ psychological distress. For descriptive analysis, we compared the prevalence of psychological distress between those who experienced childhood abuse and others, and among mothers (wives) and fathers (husbands), respectively. Then, we estimated a logistic regression model, Model 1, to predict psychological distress resulting from both parents’ experience of childhood abuse, controlling for covariates. In Model 1A, we additionally controlled for educational attainment and household income to examine the confounding effects of these socioeconomic factors on the association between childhood abuse and psychological distress.

Then, we examined children’s problem behaviour. For descriptive analysis, we compared the prevalence of children’s problem behaviour with parents’ experiences of childhood abuse. We distinguished between daughters and sons, externalizing and internalizing behaviour, and maternal and paternal childhood abuse, respectively. Then, we estimated a logistic regression model, Model 2, to examine the association between children’s problem behaviour and each parent’s childhood abuse, independent of the other parent’s experience of been abused in childhood, with adjustment for covariates. In Model 2A, we added the binary variable of parents’ psychological distress to examine the mediating effects of this factor on the impact of parents’ childhood abuse on children’s problem behaviour. In all regression models, we adjusted standard errors for the family-level nested structure. Finally, we applied mediation analysis [21, 22] to calculate the proportion of the impact that was mediated by parents’ psychological distress out of the total impact of parents’ problem behaviour.

## Results

Table 1 compares the prevalence of psychological distress with experiences of childhood abuse among parents. For both genders, those who experienced childhood abuse were more likely to report psychological distress than others (*p* < 0.001).

**Table 1:** Comparing the prevalence of psychological distress with experiences of childhood abuse among parents^a^

	Prevalence^b^ (%)	95 % CI	*p* value
Mothers			
Abused (*n* = 81)	50.6		
Not abused (*n* = 922)	25.7		
Difference	24.9	(14.8–35.0)	< .001
Fathers			
Abused (*n* = 82)	45.1		
Not abused (*n* = 921)	26.8		
Difference	18.3	(8.2–28.4)	< .001

*Note*: ^a^Not adjusted for other variables

^b^K6 scale scores ≥ 5

Table 2 shows the impact of childhood abuse on psychological distress for mothers and fathers, respectively, with these results obtained from estimating Models 1 and 1A. Consistent with the results from Table 1, Model 1 shows that adulthood psychological distress was significantly associated with one’s own experience of childhood abuse (*p* < 0.001), independent of the spouse’s experience of childhood abuse. Further, one’s psychological distress was not affected by the spouse’s childhood abuse. Model 1A reveals that educational attainment and household income did not have a strong confounding effect on these associations.

**Table 2:** Estimated impact of parents’ childhood abuse on their psychological distress^a,b^

	Model 1		Model 1A	
	OR	95 % CI	OR	95 % CI
Mothers (*n* = 1003)				
Abused in childhood	2.96***	(1.87–4.71)	3.01***	(1.89–4.80)
Spouse abused in childhood	1.40	(0.86–2.27)	1.36	(0.84–2.22)
Lower educational attainment			1.24	(0.90–1.70)
Household income poverty			1.44*	(1.01–2.07)
Fathers (*n* = 1003)				
Abused in childhood	2.26***	(1.43–3.58)	2.19***	(1.38–3.48)
Spouse abused in childhood	0.91	(0.54–1.53)	0.91	(0.54–1.53)
Lower educational attainment			1.19	(0.82–1.50)
Household income poverty			1.36	(0.97–2.06)

***p < 0.001, *p < 0.05

*Note*: ^a^K6 scale scores ≥ 5

^b^All models were controlled for children’s age group (2–3 years or 4–18 years) and the respondent of the children survey (a mother or not)

Table 3 shows the prevalence of children’s externalizing and internalizing behaviour by parental childhood abuse. Problem behaviour was more prevalent among daughters with mothers who experienced childhood abuse than the others, for both internalizing (*p* = 0.008) and, to a lesser extent, externalizing (*p* = 0.058) dimensions. In contrast, the prevalence of daughters’ problem behaviour did not differ significantly by father’s experience of childhood abuse. Among sons, both externalizing and internalizing problems were more prevalent among those whose fathers were abused in childhood than others (*p* = 0.002 and 0.003, respectively). The same pattern was observed with mothers’ childhood abuse, albeit with higher *p* values (*p* = 0.049). It should be noted, however, that these results were not adjusted for the covariates.

**Table 3:** Comparing the prevalence of children’s problem behaviour with parental childhood abuse^a^

	Externalizing			Internalizing		
	Prevalence (%)	95 % CI	*p* value	Prevalence (%)	95 % CI	*p* value
Daughters (*n* = 865)						
Mother						
Abused (*n* = 64)	10.9			10.9		
Not abused (*n* = 801)	5.2			3.9		
Difference	5.7	(−0.2–11.6)	0.058	7.1	(1.9–12.3)	0.008
Father						
Abused (*n* = 70)	7.1			7.1		
Not abused (*n* = 795)	5.5			4.2		
Difference	1.6	(−4.1–7.3)	0.577	3.0	(−2.0–8.0)	0.242
Sons (*n* = 885)						
Mother						
Abused (*n* = 68)	10.3			11.8		
Not abused (*n* = 817)	4.8			5.8		
Difference	5.5	(0.0–11.0)	0.049	6.0	(0.0–12.0)	0.049
Father						
Abused (*n* = 68)	13.2			14.7		
Not abused (*n* = 817)	4.5			5.5		
Difference	8.7	(3.2–14.2)	0.002	9.2	(3.2–15.2)	0.003

*Note*: ^a^Not adjusted for other variables

Table 4 summarizes the associations of children’s problem behaviour with parents’ childhood abuse and psychological distress, with the results obtained from estimating Models 2 and 2A. In Model 2, daughters’ externalizing and internalizing problems was significantly associated with mothers’ childhood abuse but not with fathers’. In contrast, sons’ externalizing and internalizing problems were associated with fathers’ childhood abuse only. The odds ratios of their problem behaviour in response to mothers’ childhood abuse exceeded two for both externalizing and internalizing as with the daughters, but they were non-significant due to large variances.

**Table 4:** Estimated associations of children’s problem behaviour with their parents’ childhood abuse and psychological distress^a^

	Externalizing				Internalizing			
	Model 2		Model 2A		Model 2		Model 2A	
	OR	95 % CI	OR	95 % CI	OR	95 % CI	OR	95 % CI
Daughters (*n* = 865)								
Mother: abused	2.44*	(1.04–5.71)	1.74	(0.68–4.45)	2.91*	(1.11–7.65)	1.99	(0.69–5.74)
Father: abused	1.30	(0.48–3.53)	1.06	(0.39–2.85)	1.68	(0.56–5.04)	1.22	(0.36–4.12)
Mother: psychological distress^b^			3.39***	(1.87–6.13)			5.67***	(2.53–12.7)
Father: psychological distress			1.47	(0.80–2.70)			1.46	(0.67–3.15)
Sons (*n* = 885)								
Mother: abused	2.28	(0.90–5.80)	1.83	(0.75–4.43)	2.26	(0.99–5.14)	1.95	(0.91–4.18)
Father: abused	3.18**	(1.43–7.08)	3.01**	(1.36–6.67)	2.94**	(1.43–6.05)	2.55*	(1.21–5.40)
Mother: psychological distress			3.18***	(1.77–5.70)			2.24***	(1.23–4.09)
Father: psychological distress			1.29	(0.69–2.40)			1.90*	(1.03–3.52)

***p < 0.001, **p < 0.01, *p < 0.05

*Note*: ^a^K6 scale scores ≥ 5

^b^All models were controlled for children’s age group (2–3 years or 4–18 years) and the respondent to the children survey (mother or not)

In Model 2A, we additionally adjusted for parents’ psychological distress to examine its mediating effect. The association between mothers’ childhood abuse and daughters’ externalizing and internalizing problems declined from that in Model 2 and became non-significant, while mothers’ psychological distress was positively related to problem behaviour.

In contrast, the association between fathers’ childhood abuse and sons’ externalizing and internalizing behaviour remained positive even after adding parents’ psychological distress. As with the daughters, we found that mothers’ psychological distress had a positive association with sons’ problem behaviour. Fathers’ psychological distress had a significant association with sons’ externalizing behaviour, but not with their internalizing behaviour.

Table 5 presents the proportions of the impact mediated by parents’ psychological distress out of the total impact of parents’ childhood abuse on children’s problem behaviour for daughters and sons. We concentrated on the impact of mothers’ childhood abuse for daughters and that of fathers’ childhood abuse for sons, respectively, because they were shown to be significant in Table 4. For daughters’ problem behaviour, the total impact mediated by both parents’ psychological distress was mostly attributable to mothers’ psychological distress. For sons, the total impact mediated by both parents was much smaller than that for daughters, and the mediation effect of either parent’s psychological distress was not significant for externalizing behaviour.

**Table 5:** Estimated proportions of the impact mediated by parents’ psychological distress^a,b^

	Externalizing		Internalizing	
	Proportion (%)	95 % CI^c^	Proportion (%)	95 % CI
Daughters (*n* = 865)				
The impact of mothers’ childhood abuse mediated by				
Mother’s psychological distress	37.9^d^	(16.3–67.7)	41.0^d^	(19.5–72.2)
Father’s psychological distress	−1.8	(−17.9–3.9)	−1.4	(−16.3–3.2)
Total	36.1^d^	(10.0–66.9)	39.7^d^	(13.8–70.0)
Sons (*n* = 885)				
The impact of fathers’ childhood abuse mediated by				
Mother’s psychological distress	7.2	(−4.4–23.9)	5.5	(−2.5–20.1)
Father’s psychological distress	4.4	(−6.6–16.8)	12.1^d^	(1.1–29.9)
Total	11.6	(−4.7–30.9)	17.6^d^	(3.4–39.1)

*Note*: ^a^K6 scale scores ≥ 5

^b^Calculated based on the estimation results in Models 1, 1A, 2, and 2A. Fathers’ and mothers’ experiences of childhood abuse were used as covariates in estimations for daughters and sons

^c^Bias-corrected and accelerated confidence interval obtained by bootstrap estimations (with 2000 iterations), given the point-estimated total impact of parents’ childhood abuse on children’s problem behaviour

^d^indicates that the 95 % CI does not include zero.

## Discussion

We have examined the linkages between parents’ experience of physical abuse and neglect and children’s problem behaviour, with special reference to gender-specific features. We used household data for parents and their child(ren), which made it possible to consistently investigate the intergenerational effect of child abuse and associated gender differences. Before examining the intergenerational impact of childhood abuse, we confirmed that each parent’s psychological distress was reliably predicted by his/her experience of having been abused in childhood, which is generally consistent with the findings in previous studies [5–8]. In addition, we found that only a small portion of this association was confounded by adulthood socioeconomic status [7].

More important, the results highlighted the gender-specific aspects of the associations between parents’ childhood abuse and their children’s problem behaviour. First, we observed strong mother–daughter and father–son linkages. Daughters’ problem behaviour was more closely associated with their mothers’ childhood abuse than with their fathers’, whereas sons’ problem behaviour was more closely associated with their fathers’ experience. The odds of sons’ problem behaviour in response to mothers’ childhood abuse were relatively high, but their association was non-significant. These gender-specific results are generally consistent with what the theory of same-gender dyads suggests; parents’ psychological and behavioural properties would have a greater influence on the development of children of the same gender [12, 13]. Existing literature in child psychology also supported this theory based on findings that children’s delinquent behaviours had a stronger association with the parenting style of the same-gender parent [23]; however, another study revealed contradicting findings [24]. Further accumulation of empirical evidence is therefore needed to examine the applicability of the theory to the linkage between parental childhood abuse and child’s problem behaviours.

Second, we found that the association between parental childhood abuse and children’s problem behaviour remained significant only between fathers and sons, if controlling for parents’ psychological distress. This observation is supportive of a view that the effect of negative parenting style, which is associated with childhood abused experience [25–27], is stronger in father–son pairs than in other parent–child pairs, as found in previous empirical studies on parenting style [23, 28]. Since few studies examined the effect of fathers on children’s problem behaviours, accumulation of evidence is needed to confirm that the intergenerational effect of childhood abuse is magnified when parent and children have the same gender.

Third and related to the second point, we found a remarkable difference in the mediating effect of parents’ psychological distress between daughters and sons. The impact of mothers’ childhood abuse on daughters’ problem behaviour was partially mediated by mothers’ psychological distress. In contrast, neither parents’ psychological distress had much effect on the association between fathers’ childhood abuse and sons’ problem behaviour. We confirmed that sons’ problem behaviour was closely associated with mothers’ psychological distress; however, mothers’ psychological distress was not affected by their spouse’s childhood abuse, meaning that mothers’ psychological distress did not mediate the impact of fathers’ childhood abuse on sons’ problem behaviour either.

These findings suggest the relatively limited mediating effect of parents’ psychological distress on the impact of parents’ childhood abuse on children’s problem behaviour especially among boys. Consistently, we observed in the current study that the impact of fathers’ childhood abuse on sons’ problem behaviour was mediated at a rate of less than 20 % by both parents’ psychological distress, while the proportion of the mediating effect was about 40 % in mother–daughter pairs. The limited mediating effect implies the contribution of other possible mediating factors, such as parents’ aggression towards children and maltreatment [24], insecure attachment style [29, 30], or substance abuse [2], although they are likely to have close associations with parents’ psychological distress. Further investigation is required to explain the gender difference in the mechanisms of intergenerational effect of parental childhood abuse.

Besides these gender differences, we found that both sons’ and daughters’ problem behaviour was much more closely associated with mothers’ psychological distress than fathers’. This finding is in accordance with existing literature that found a greater role of mothers, than fathers, in child development [31]. Fathers’ less frequent contact with children may limit the effect of fathers’ psychological distress on children; indeed, time spent with children among mothers was 6.5 times as long as the time fathers spent with children in the current sample.

We recognize that the current study has some important limitations. First, the J-SHINE did not measure sexual abuse, because it was believed to trigger negative reaction to this study due to the stigma and embarrassment attached to sexual victimization in Japan [32]. Psychological abuse was not measured either, because of the difficulty in assessing childhood psychological abuse based on retrospective self-reports. Violence from significant others (i.e., family and intimate partners) is often subject to recall bias [33, 34], and there may be a greater risk of reporting psychological abuse inconsistently [27]. Although psychological abuse is often accompanied with physical abuse and neglect [35], the lack of assessment of psychological abuse may have underestimated the association of parental childhood abuse with the child’s problem behaviours in this study.

Second, we were not able to distinguish legally defined child physical abuse from corporal punishment. The Child Abuse Prevention Act in Japan defines child physical abuse as a type of violence that actually or potentially causes injury to children, and the mere use of physical force is not legally prohibited in Japan. We were not able to identify physical abuse in the legal context, because the J-SHINE did not measure actual injury or threat of injury as a result of physically injurious disciplining. Although the use of physical force in parenting may eventually result in physical abuse, the actual association of physical abuse and child’s problem behaviours may have been underestimated because of the broader definition of physical abuse utilized in this study.

Third, the study sample was collected from four communities in Tokyo metropolitan area. The limited representativeness, together with a relatively small sample size, necessitates caution in generalizing the estimation results. Forth, the cross-sectional nature of the study design means that we could not precisely identify causation between variables. While it is reasonable to examine the one-way causality from parents’ childhood abuse to children’s problem behaviour, parents’ psychological distress and children’s problem behaviour are likely to be affected by each other [36]. Fifth, retrospective reports of adverse childhood experiences in this study are likely to involve measurement errors. We cannot rule out the possibility that these reports were affected by the participants’ current mental health [37]. For example, a current depressive state could lead one’s attention to painful memories associated with childhood abuse, while improved mental health may result in reconceptualizing the abuse experience as non-threatening. However, it was also suggested that a depressive mental state could overly generalize one’s negative experiences in childhood [37]. The direction of effect of the current mental health status on measurement errors is yet to be explored.

## Conclusions

Despite these limitations, the current study underscores that the intergenerational impact of parental childhood abuse on children’s problem behaviour is gender specific, i.e., largely characterized by the same gender linkages. The findings in the current study also suggest the mediating effect of parents’ psychological distress on the impact of parents’ childhood abuse on children’s problem behaviour is relatively limited among boys. Further studies that explore the mechanisms involved in the intergenerational impact of childhood abuse are needed.

## Ethics and consent to participate

The Research Ethics Committee of the University of Tokyo, Graduate School of Medicine approved the survey procedure of the J-SHINE (Japanese Study of Stratification, Health, Income, and Neighborhood) (No. 3073-[1]). The questionnaire was computer-assisted and self-administered, unless participants requested a face-to-face interview. Participation in this study was voluntary, and written consent was obtained from each respondent. The Research Ethics Committee of the University of Tokyo, Graduate School of Medicine granted approval for the authors to use the dataset, because they belonged to the members who designed and conducted J-SHINE.

## Consent to publish

There are no details on individual participants within the manuscript.

## Availability of data and materials

The dataset of J-SHINE used for this study is not openly available, because the Research Ethics Committee of the University of Tokyo, Graduate School of Medicine prohibits researchers from providing their research data to other third-party individuals.

## Abbreviations

CBCL: child behavior checklist
J-SHINE: Japanese Study of Stratification, Health, Income, and Neighborhood
K6: Kessler Psychological Distress Scale
