# Erratum to: gender-specific linkages of parents’ childhood physical abuse and neglect with children’s problem behaviour: evidence from Japan

**DOI:** 10.1186/s12889-017-4571-6

**Published:** 2017-07-12

**Authors:** Takashi Oshio, Maki Umeda

**Affiliations:** 1 Institute of Economic Research, Hitotsubashi University, 2-1 Naka, Kunitachi-shi, Tokyo, 186-8603 Japan; 2 Graduate School of Nursing Science, St. Luke’s International University, 10-1 Akashi-cho, Chuo-ku, Tokyo, 104-0044 Japan

## Erratum

After publication of the article [1], it has been brought to our attention that the information given regarding the availability of data and materials is incorrect. The section should read “The use of J-SHINE data is to be approved by the data control committee of the J-SHINE researcher group by request”.
